# Contact Screening and Isoniazid Preventive Therapy Initiation for Under-Five Children among Pulmonary Tuberculosis-Positive Patients in Bahir Dar Special Zone, Northwest Ethiopia: A Cross-Sectional Study

**DOI:** 10.1155/2020/6734675

**Published:** 2020-06-05

**Authors:** Netsanet Fentahun, Yosef Wasihun, Abebe Mamo, Lakew Abebe Gebretsadik

**Affiliations:** ^1^School of Public Health, College of Medicine and Health Science, Bahir Dar University, Bahir Dar, Ethiopia; ^2^Department of Health, Behavior and Society, Institutes of Health, Jimma University, Jimma, Ethiopia

## Abstract

**Background:**

Children are highly susceptible to *Mycobacterium tuberculosis* infection, and about 70% of children living in the same households with pulmonary tuberculosis-positive patients will become infected. However, pulmonary positive tuberculosis is a common phenomenon and the implementation of the recommended contact screening and initiation of isoniazid preventive therapy is very low. Therefore, this study is aimed at assessing contact screening practice and initiation of isoniazid preventive therapy of under-five children among pulmonary tuberculosis-positive patients in Bahir Dar, northwest Ethiopia.

**Methods:**

A facility-based cross-sectional study was conducted from March 1 to 30, 2016. A total of 267 pulmonary tuberculosis-positive patients were included in this study. To identify independent predictors of contact screening and isoniazid preventive therapy initiation, we performed multivariable logistic regression analyses using SPSS version 20 with CI of 95% at *p* value < 0.05.

**Results:**

A total of 230 (90.2%) pulmonary tuberculosis-positive patients had single contacts with their under-five children. One hundred nine (64.8%) children were screened. From those screened, 11 (7.4%) developed tuberculosis disease and started antituberculosis treatment. Forty-four (31.9%) children started isoniazid preventive therapy. Sex of the participants, place of service delivery, relationship with contacts, HIV status, and attitude of PTB+ cases were significant predictors of contact screening (*p* < .05). Participant's knowledge, attitude of participants, and relationship of the child with participant were significant predictors of isoniazid preventive therapy initiation (*p* < 0.05).

**Conclusion:**

Contact screening practice and isoniazid preventive therapy initiation of children under the age of 5 in Bahir Dar zone were very low. Intimate family contact with pulmonary tuberculosis-positive patients, place of service delivery, and attitude towards screening are the key factors of contact screening. Participant's knowledge, attitude of participants, and relationship of the child with participant are the key factors of isoniazid preventive therapy initiation. Therefore, household contact screening and isoniazid preventive therapy initiation should be paid attention to reduce transmission.

## 1. Introduction

Tuberculosis (TB) is a chronic necrotizing disease caused by Mycobacterium tuberculosis complex. The species commonly involved are *M. tuberculosis*, *M. bovis*, *M. africanum*, and *M. microti* [1]. Worldwide, an estimated 10 million people fall ill with TB and about 1.5 million cases died in 2018. From these 10 million cases, about 1.1 million (11%) occurred in children (0-14 years) and there were 205,000 child deaths due to TB in 2018 [2]. The World Health Organization (WHO) reported that a number of new TB cases occurred in Southeast Asia (44%), Africa (24%), and Western Pacific (18%) globally in 2018 [2]. Almost 90% of cases each year are in 30 high TB burden countries, and eight countries account for two-thirds of the total [3].

In Ethiopia, TB is one of the leading infectious diseases, and in 2014/2015, 135,831 TB cases (all forms) were reported; of these, 35% were bacteriologically confirmed pulmonary TB cases [4].

TB ranks among the top causes of childhood morbidity and mortality in developing countries but neglected for years in children [5]. Children living in close contact with PTB+ are at high risk of TB infection. One cough can produce 3,000 and a sneeze up to a million droplet nuclei in which the infectious dose of TB is 1 to 10 bacilli [6].

Contact screening and providing treatment are important and a simple strategy to implement at the primary health care setting [7]. Various studies demonstrated screening as an effective strategy in early identification of children eligible for isoniazid preventive therapy (IPT) and preventing susceptible children from developing the disease who had household contacts with PTB+ patients [8–10]. WHO and CDC reported that IPT is effective for about 93% of under-five children and 59% among children aged 15 years or younger and IPT is recommended in a daily basis for at least 6 months [11–13].

Rates for the initiation of IPT in eligible children ranging from 1.3% to 26% are reported in settings where tuberculosis is endemic [14]. High-burden countries have reported extremely poor compliance with screening and initiation of IPT because of limited awareness of its benefits and inability to perform screening tests by health care providers. Interruption of INH supply and distance from health facilities affect uptake of the service in different settings [15].

Contact tracing, investigation, and prophylaxis of childhood contacts of adult TB cases are widely recommended but rarely practiced in developing countries [16]. Ethiopia is also one of the 22 high-burden countries for TB, and childhood TB is still a major cause of hospital admission and death [17]. In 2014, of the new cases notified in Ethiopia, fourteen percent were pediatric TB cases. Even this could be an underestimate due to difficulty in confirmation of diagnosis of TB in children [18]. Ethiopia has accepted and implemented the WHO's recommendation of a six-month course of IPT as one strategy for prevention, care, and control of TB [19]. Ethiopia also developed a national TB prevention and treatment guideline which emphasizes on contact screening and IPT for under-five children who have a history of contact with PTB+ patients. Clinical assessment alone is conducted to decide whether the contact is eligible for IPT initiation or not. But for symptomatic contacts, further diagnosis like chest radiograph and ESR is conducted to rule out active TB [8, 20].

However, it has been evident that the implementation of the recommended contact screening is very low and IPT initiation has been largely ignored in Ethiopia [8, 15]. In addition, there is limited information regarding suboptimal contact screening and noninitiation of IPT. These indicated that there is a huge gap in implementing the recommended strategies. Hence, identifying and understanding the factors that affect the implementation of contact screening and IPT initiation are crucial to address the challenges. Therefore, the objective of this study was to assess the contact screening and INH prophylaxis practices of under-five children among PTB+ patients in Bahir Dar special zone, northwest Ethiopia.

## 2. Methods and Materials

### 2.1. Study Design and Settings

This study used a facility-based cross-sectional study design to assess household contact screening and INH prophylaxis of under-five children who had contact with PTB+. We conducted this study in Bahir Dar special administration zone, with 221,991 predominantly urban populations, Amhara regional state of Ethiopia. It is located around 565 km northwest of Addis Ababa, the capital of Ethiopia. In this zone, there were around 989 TB patients of all forms and 460 were PTB+ patients. The special zone has 18 governmental health facilities (4 hospitals and 14 health centers), which were giving directly observed treatment (DOT) services in the first quarter of 2016.

### 2.2. Study Participants and Procedures

The study population was all PTB+ patients who had under-five children and registered their household contacts during November 2015–March 2016. These participants were interviewed during DOTS program at health facilities from March 1 to 30, 2016. As per guidelines [8], all diagnosed PTB+ patients are expected to be recorded in the contact investigation register. So, we used the data routinely recorded in the contact investigation register at all health facilities (4 hospitals and 14 health centers) providing DOT services in the study areas. From these health facilities, there were around 460 PTB+ patients, and 267 of them were listed on TB treatment registration book as PTB+ patients who had under-five children and we included all 267 of them as census. Every day, we gathered all PTB+ patients in the morning and selected those who had under-five children at home for interview. Patients' TB treatment cards were used for identification of those PTB+ patients. During data collection, those children who were screened and started IPT and anti-TB treatment were cross-checked from IPT and anti-TB treatment registration books, respectively.

The Ethiopian National TB Program recommends the following groups of persons to be given priority for identification through contact tracing and undergo clinical evaluation: symptomatic contact of the index patient, contacts who are living with HIV, contacts who are under-five children, and contact of an index case with presumed/confirmed DR-TB. Contact tracing is initiated by the TB focal person as soon as the index case is registered to receive TB treatment. The TB focal person communicates with the health extension workers to conduct identification of contacts at the household/community level, do symptom-based TB screening, and refer those who require detailed evaluation and investigation at the health facility level. The initial identification and evaluation of identified contacts for active TB are conducted using symptom-based TB screening questions and may be conducted at the community level by health extension workers and/or at a health facility by trained health care workers.

**Figure 1:**
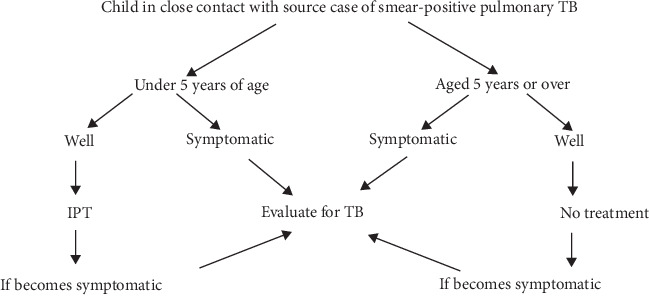
Approach contact management of children in Ethiopia.

In Ethiopia, we have two modalities for TB contact tracing. The first modality is contact tracing at the community level: as part of the community TB care package, health extension workers are practicing household contact tracing during routine home visits and upon receiving the request from the catchment health facility. The health extension workers are preferred to identify those at high risk of developing TB and symptomatic ones and bring them to the health facility for clinical evaluation and subsequent management. The second modality is contact tracing at the health facility level: the health care worker at the TB clinic should initiate contact tracing and investigation for close/household contacts upon registering a new index TB patient for treatment (Figure 1).

### 2.3. Data Collection and Measurements

PTB+ patients with child contacts were interviewed in Amharic (local language) using a pretested, structured, and interviewer-administered questionnaire. The questionnaire is developed by reviewing different literatures [21–24] and prepared in English first and translated into the local language. The questionnaire included sociodemographic variables, knowledge, and attitude of PTB+ patients on tuberculosis, contact screening, and INH prophylaxis and health worker-related questions. The questionnaire was pretested in Merawi district which is found 32 km far from the study area. The data were collected by four college completed BSc nurses and one health officer as a supervisor. Training was given for data collectors about the data collection tool, how to collect data, and taking consent to have common understanding.

The knowledge question was assessed using different items including sign and symptoms, cause, mode of transmission, risk groups, prevention mechanism, and treatment of tuberculosis. Study participants were interviewed on all mentioned dimensions regarding tuberculosis with “yes or no” questions, and finally, a composite measure for each aspect was created out of 15 and this weighted score was used for analysis as continuous variable.

The attitude part of the questions consisted of five items in five-point Likert scale. The scores of individuals for each item were summed up and ranged from 5 minimum to 25 maximum total score after reverse coding for negatively worded items, and this was treated as continuous variable for analysis. Reliability test was checked using Cronbach's alpha and the results were 0.87 and 0.74, respectively.

Contact screening was assessed using two items with the responses of “Yes or No” type like “have you ever heard or been informed about contact screening”? Those who responded “Yes” were considered as eligible for the next question and were asked “have your under-five child/children been screened for TB”? Those who responded “Yes” were considered as they were practicing contact screening. Finally, children who were screened and found free from TB were identified and considered as eligible for IPT and assessed using an item, “did your child/children start INH prophylaxis”? Those who responded “Yes” were considered as the children were starting IPT.

### 2.4. Data Analysis

Data were coded and entered to a computer using EpiData software version 3.1 (EpiData Association, Odense, Denmark) and exported to SPSS program version 20.0 for further analysis. The descriptive result was presented using frequency and proportions for all variables, and we performed bivariate regression analysis to determine association between factors of contact screening and IPT initiation at 95% confidence interval and *p* value of <0.25. We performed multivariate logistic regressions, and variables with *p* value < 0.05 are considered to identify the independent predictors of contact screening and IPT initiation. Goodness of fit of the final models was checked using Hosmer and Lemeshow test of goodness fit for contact screening and IPT, and the results were 0.86 and 0.26, respectively.

## 3. Result

### 3.1. Sociodemographic Characteristics

Overall, 267 PTB+ patients who had household contacts were registered between November 2015 and March 2016. A total of 255 PTB+ patients were interviewed from March 1 to 30, 2016, with a response rate of 95.5%. From the interviewed PTB+ patients, 131 (51.4%) were males, 153 (60%) of them were parents (father or mother) of the contacts, and more than half of the respondents 134 (52.5%) were married. Other sociodemographic characteristics of respondents and related factors are given in Table 1.

### 3.2. Health Information Related to Contact Screening and IPT Initiation

From the 255 interviewed PTB+ patients, 230 (90.2%) of them informed having under-five contacts (1 under-five contact/PTB+ patient), for a total of 230 under-five children evaluated. Of these 230 under-five children, 149 (64.8%) of them were screened for active TB and 11 (7.4%) were diagnosed with TB disease and started anti-TB treatment. One hundred thirty-eight child contacts who were diagnosed as free from TB disease were considered as eligible for IPT; of whom, 44 (31.9%) started IPT. The mean score of knowledge and attitude was 9.74 (SD ± 3.94) and 17.81 (SD ± 2.59), respectively. Contact screening, initiation of IPT, and other related factors are given in Table 2.

### 3.3. Factors Associated with Household Contact Screening of Under-Five Children

After controlling for possible confounding factors through multiple logistic regressions, place of service provided, relationship of the index case with the contact, and attitude of respondents were independent predictors of contact screening. Females who had contact with children were five times more likely to bring their under-five children for contact screening than males (AOR = 5.3, 95% CI (1.2, 23.2)). Participants who received the services from a hospital were 7 times more likely to bring their under-five children for contact screening as compared to those participants who received the services from a health center (AOR = 6.5, 95% CI (1.2, 41.8)). Parents who had a contact with children were 15 times more likely to bring their under-five children for contact screening as compared to siblings (brothers, sisters, and other relatives) (AOR = 14.8, 95% CI (3.2, 69.7)). HIV-positive TB patients were 19 times more likely to bring their under-five children for contact screening as compared to HIV-negative TB patients (AOR = 19, 95% CI (2.1, 16.87)). A unit increase in total score of attitude of the participants in the odds of household contact screening was increased by 2.8 (AOR = 2.8, 95% CI (1.5, 5.2)) (Table 3).

### 3.4. Factors Associated with Initiation of IPT for Under-Five Children

Multiple logistic regression analysis was performed to identify independent predictors of IPT initiation. Children who have parents as index case were twenty times more likely to start IPT as compared to children who had siblings (AOR = 20.0, 95% CI (2.4, 168.1)). A unit increases of attitude of participants; the odds of IPT initiation is increased by 3.4 (AOR, 95% CI 3.4 (1.1, 10.2)). A unit increases of knowledge of the participants; the odds of starting IPT initiation is increased by 2.9 (AOR, 95% CI 2.9 (1.6, 5.2)) (Table 4).

## 4. Discussion

This study provides insight into the operation of household contact screening and IPT initiation of under-five children in northwest Ethiopia. About 64.8% of child contacts underwent screening for TB in our study, and other studies from Vietnam [25], Malawi [26], and Thailand [27] reported much less screening rates (6-52%), while a study in Western Cape Province of South Africa reported much higher screening rates (91%) [28]. The possible explanation for these differences could be due to the lack of an extensive awareness campaign about TB and its treatment, passive contact screening method, and lack of follow-up of the index cases to bring their under-five children to health facilities by health workers.

In addition to contact screening, this study also found that only 31.9% of children were started with IPT. This finding is lower than a study reported in Indonesia which reported 40% of IPT initiation [29], much lower than a study in India (56.4%) [30], but higher than other study in India (19%) of IPT initiation [21]. Inaccessibility of health facilities, isoniazid stock-outs, and risk perception of participants to start IPT without any sign and symptoms could be the main justifications for low initiation of IPT in our study.

In this study, contact screening and initiation of IPT were more likely among child contacts where index case is parents than siblings. This is consistent with the study findings from Vietnam [25] and Timor Leste [31]. In India, children living with parents were more likely to have initiated on IPT than other household members [32]. This study implies that parents are more concerned for their children's health and well-being and the bond between parents and small children are stronger than other household members. These reasons have also been reported in a study from Thailand [27].

This study also found that household contact screening of under-five children was more likely among hospital users than health center users. Studies from Thailand and Malawi reported that health center-based contact screening was poorly used because different types of laboratory services and tests were not available in the health centers, so the hospital is identified as a better option [27, 33].

This study showed that HIV-positive patients had significant association with contact screening unlike a previous study [22]. This could be because HIV-positive patients had regular visits to a health facility for antiretroviral therapy follow-up which might in turn made them know the benefit of early contact screening than HIV-uninfected patients.

Attitude of tuberculosis patients towards household contact screening without signs and symptoms and starting IPT in eligible under-five children had significant association with contact screening as well as IPT utilization. This finding was coherent with a study in India which revealed that most TB patients like to use screening and initiate IPT and they believed that the use of IPT has contributed to a higher reduction of acquiring tuberculosis [33]. This implies that if people believe in positive outcome, they accept it without trying to prove that the treatment is working or not.

According to this study, we found that knowledge was a major influencing factor for contact screening of under-five children. Per a unit increase in total score of knowledge on preventive mechanisms of TB, the odds of IPT initiation is also increased. A study in Ethiopia explained that 79.3% responded transmission of TB would be preventable and 80% knew that TB can be transmitted from a patient to another person [18]. Different studies justified that high-burden countries have reported extremely poor compliance with initiation of IPT because of limited awareness of its benefits and inability to perform prerequisite screening tests.

Rates for the initiation of IPT in eligible children ranging from 1.3 to 26% have been reported in settings where tuberculosis is endemic [14, 22]. As explained in a health belief model, when the barriers of health action outweigh the benefits in the minds of a given person, the likelihood of taking action decreases [33]. Therefore, certain types of barriers are more or less important for particular cultures or norms. Thus, it is not only knowledge but also attitude and other factors which could be special factors associated with the initiation of IPT among participants. As we conducted this study, there were a few limitations, which relate to the use of self-reported questions on knowledge of patients on tuberculosis, contact screening, and INH prophylaxis, which could lead to social desirability biases. However, as these questions were validated in a similar population, it is expected that this problem is minimal. Again, this study is conducted in one zone of the Amhara region. Whether the findings from this administrative zone are representative of contact screening and IPT of the whole region is unknown. But this special administrative zone has relatively higher number of population than other zones.

## 5. Conclusion

Household contact screening of under-five children and initiation of IPT in this study were very low. Sex of the participants, place of service delivery, relationship with contacts, HIV status, and attitude of PTB+ cases are the key factors for contact screening. Knowledge of participants, attitude of participants, and relationship with contacts are the key factors of initiation of IPT. The regional health bureau and health facilities should pay attention on contact screening and IPT initiation to narrow the practice gaps in Ethiopia.

## Figures and Tables

**Table 1:** Sociodemographic and related profile of PTB+ patients in Bahir Dar special zone, northwest Ethiopia, March 2016 (*N* = 255).

Variables	Categories	Frequency	Percent
Age in years	<20	36	14.1
	20-29	89	34.9
	30-39	84	32.9
	40-49	32	12.5
	>49	14	5.5

Sex	Male	131	51.4
	Female	124	48.6

Religion	Christian	239	93.7
	Muslim	16	6.3

Residence	Urban	168	65.9
	Rural	87	34.1

Marital status	Married	134	52.5
	Unmarried	101	39.6
	Divorced	7	2.7
	Widowed	13	5.1

Educational status	No formal education	75	29.4
	Primary school educated	100	39.2
	Secondary school educated	71	27.8
	College/university educated	9	3.5

Occupation	Farmer	50	19.6
	Merchant	41	16.1
	Student	36	14.1
	Housewife	32	12.5
	Daily laborer	27	10.6
	Government employee	20	7.8
	No work	26	10.2
	Driver	23	9

Household income	<500 eth birr	106	41.6
	500-1000 eth birr	83	32.5
	>1,000 eth birr	66	25.9

Relationship of the index cases with contacts	Parents	153	60
	Siblings	102	40

**Table 2:** Health information related to contact screening and IPT initiation of PTB+ patients in Bahir Dar special zone, northwest Ethiopia, March 2016 (*N* = 255).

Variable	Category	Frequency	Percent
Informed about contact screening	Yes	230	90.2
	No	25	9.8
Practice contact screening (*N* = 230)	Yes	149	64.8
	No	81	35.2
Outcome of contact screening	Positive	11	7.4
	Negative	138	92.6
Informed about the presence of IPT	Yes	82	32.2
	No	173	67.8
Started to use IPT (*N* = 138)	Yes	44	31.9
	No	94	68.1
HIV status (*N* = 230)	Positive	34	14.8
	Negative	196	85.2
Place of service provision (*N* = 230)	Health center	190	82.6
	Hospital	40	17.4

IPT: isoniazid preventive therapy; PTB+: smear-positive pulmonary tuberculosis; HIV: human immuno virus.

**Table 3:** Factors associated with household contact screening of under-five children, about contact screening in Bahir Dar special zone, northwest Ethiopia, March 2016 (*N* = 230).

Variables		Contact screening		COR (95% CI)	AOR (95% CI)
		Yes	No		
Sex	Male	69 (46.3%)	50 (61.7%)	1	1
	Female	80 (53.7%)	31 (38.3%)	1.9 (1.1, 3.2)	**5.3 (1.2, 23.2)**∗
Residence	Rural	29 (19.5%)	40 (49.4%)	1	1
	Urban	120 (80.5%)	41 (50.6%)	4.1 (2.2, 7.3)	1.02 (0.2, 5.5)
Health facility	Health center	114 (76.5%)	76 (93.8%)	1	1
	Hospital	35 (23.5%)	5 (6.7%)	4.7 (1.8, 12.4)	**6.5 (1.2, 41.8)**∗
HIV status	Negative	119 (79.9%)	77 (95.1)	1	1
	Positive	30 (20.1%)	4 (4.9%)	4.9 (1.6, 14.3)	**19 (2.1, 16.87)**∗∗
Relationship of the child with participant	Parents	107 (71.8%)	34 (42%)	3.5 (2, 6.2)	**14.8 (3.2, 69.7)**∗∗
	Sibling^#^	42 (28.2%)	47 (58%)	1	1
∗∗^#^Knowledge score				1.7 (1.5, 1.9)	0.9 (0.6, 1.3)
∗∗^#^Attitude score				3.1 (2.3, 4.2)	**2.8 (1.5, 5.2)**∗∗

∗Continuous variables. AOR: adjusted odds ratio; COR: crude odds ratio; HIV: human immuno virus. ^#^Sibling: brothers, sisters, and relatives.

**Table 4:** Factors associated with initiation of IPT of under-five children, in Bahir Dar special zone, northwest Ethiopia, March 2016 (*N* = 138).

Variable		IPT prophylaxis		COR with 95% CI	AOR with 95% CI
		Started	Not started		
Relationship of the case with contacts	Siblings	2 (4.5%)	37 (39.4%)	1	1
	Parent	42 (95.5%)	57 (60.6%)	13.6 (3.1, 59.7)	**20.0 (2.4, 168.1)**
Age of the child	<1 year	3 (6.8%)	40 (42.6%)	0.07 (0.02, 0.3)	1.9 (0.3, 11.2)
	1-2 years	11 (25%)	26 (27.7%)	0.4 (0.2, 1.0)	1.8 (0.3, 11.2)
	>2 years	30 (68.2%)	28 (29.8%)	1	1
HIV status	Negative	25 (56.8%)	86 (91.5%)	1	1
	Positive	19 (43.2%)	8 (8.5%)	8.2 (3.2, 20.9)	0.4 (0.5, 1.5)
∗Attitude	*β* = 1.230			6.8 (3.2, 14.5)	**3.4 (1.1, 10.2)**
∗Knowledge	*β* = 1.065			3.1 (1.9, 4.9)	**2.9 (1.6, 5.2)**

∗Continuous variables. AOR: adjusted odds ratio; COR: crude odds ratio; IPT: isoniazid preventive therapy.

## Data Availability

The datasets supporting the conclusions of this article are included within the article.

## Abbreviations

AOR: Adjusted odds ratio
CI: Confidence interval
COR: Crude odds ratio
HIV: Human immuno virus
INH: Isoniazid
IPT: Isoniazid preventive therapy
PTB+: Smear-positive pulmonary tuberculosis
TB: Tuberculosis
SD: Standard deviation
SPSS: Statistical Package for the Social Sciences
WHO: World Health Organization.
